# The safe zone of distal fibula was determined based on the classification of lateral malleolus fossa

**DOI:** 10.1186/s13018-023-04194-6

**Published:** 2023-09-22

**Authors:** Gui-xuan You, Lei Huang, Ming-hui Li, Bin Xiong, Wan-lin Peng, Hou-yin Shi, Lei Zhang

**Affiliations:** 1 School of Physical Education, Southwest Medical University, Luzhou, 646000 China; 2 School of Clinical Medicine, Southwest Medical University, Luzhou, 646000 China; 3 Department of Medical Imaging, Southwest Medical University, Luzhou, 646000 China; 4 Department of Orthopedics, The Affiliated Traditional Chinese Medicine Hospital of Southwest Medical University, Luzhou, 646000 China; 5 Center for Orthopedic Diseases Research, The Affiliated Traditional Chinese Medicine Hospital of Southwest Medical University, Luzhou, 646000 China

**Keywords:** Lateral malleolar fossa, Fibular fracture, Internal fixation, Ankle

## Abstract

**Background:**

Lateral malleolus fractures are very common, and the distal fibular geometry is complex. This study aimed to classify the lateral malleolus fossa (MF) into different types by characterizing the lateral MF imaging morphology and exploring the relationship between the lateral MF and internal fixation position after distal fibula fractures.

**Methods:**

Anteroposterior CT reconstruction was performed on 248 subjects. After reconstruction, the deepest point of the lateral MF was located, and then, the cross-sectional shape of the lateral MF was observed and classified.

**Results:**

According to the morphology of the CT cross section, the lateral MF was divided into three types: type C (43.1%), type V (32.2%), and type Flat (24.7%). Type V (3.98 ± 0.82) was significantly longer than type C(2.83 ± 0.54) and type Flat (1.84 ± 0.42) in cd. Similarly, in ∠α, Type Flat(136.31 ± 9.63) was the largest, followed by type C (116.51 ± 8.79), and type V (89.31 ± 9.07) was the smallest. Other measurements were not found any significant differences between the above.

**Conclusion:**

According to the morphology of the CT cross section, the lateral MF was divided into three types: type C, type V and type Flat. Type V is most likely to be invaded when fixing the distal fibula. Screws less than 9 mm should be selected when fixing, and screws no more than 10 mm should be selected when there are type C and type Flat of MF.

## Background

The fibula is a long bone in the lower extremity that is positioned on the lateral side of the tibia. The distal end of the fibula forms the lateral malleolus which articulates with the lateral talus, creating part of the lateral ankle [1]. The lateral malleolus fossa (MF) is located in the posteromedial part of the distal fibula and is the insertion point of the posterior talofibular ligament [2]. In addition, the lateral MF is also the anatomical structure of the posterolateral talus and subtalar joint motion space [3].

Ankle fractures account for approximately 20% of extremity fractures; most ankle fractures involve the fibula. Isolated fibula fractures accounted for 55–70% of ankle fractures, bimalleolar fractures 4–20%, and trimalleolar fractures 10–11% [4–7]. Ankle fracture instability usually requires surgical treatment. In recent years, the main methods of internal fixation have included posterolateral plate fixation, locking plate construction, intramedullary instruments, etc. [8–10] No matter which internal fixation method is selected, most of the lateral malleolus fractures will pass through the distal fibula. Many scholars have explored the safe range of fibula intramedullary nail fixation and analyzed the shape, diameter, and length of the tip of the fibula [11, 12]. At the same time, the anatomical study of the lateral MF can make the placement of ligament binding screws or/and fibular plates more reasonable [11]. Furthermore, some scholars judge whether the distal fibula fracture was reduced by evaluating the fibular notch [13]. There is evidence that the geometry of the distal fibula is complex because there are many ligaments attached to the anterolateral side, and there are many studies on the anatomical structure of the innominate tuberosity of the fibula, the anterior tuberosity of the fibula and the tip of the fibula [2, 14, 15]. When fixing the lateral malleolus fracture, we found that the morphological characteristics of the lateral MF are unique. In this context, understanding the anatomical structure of the MF has a particular reference value for the safe placement of internal fixation.

Our hypothesis is that computed tomography (CT) would help to understand the morphology of the distal fibula better. By observing CT three-dimensional reconstruction, we can more fully understand the anatomical characteristics of the lateral malleolus. In this study, the primary objective was to analyze the lateral MF, focusing on the shape of the lateral MF and its anatomical classification. The secondary objective was to describe the safe area of distal fibula internal fixation according to the anatomical classification of lateral MF.

## Methods

All ankle joint CT scan images were collected from the hospital, and Ethical approval was obtained. A total of 248 subjects were included in the study, including 107 females and 141 males.

### Patient selection

Inclusion criteria: (1) 18–70 years old; (2) CT images of the distal fibula and the lateral MF; (3) distal fibula fractures; (4) ankle deformities.

Exclusion criteria: (1) tibiofibular syndesmosis injury; (2) distal fibula fractures; (3) ankle deformities.

### Image acquisition

The German Siemens SMATOM Definitim Edge 64-slice spiral CT machine was used to scan the ankle joint of the included population. Scanning parameters: tube voltage 120 k V, tube current 280 m A, scanning angle 0, layer thickness 1.25 mm, layer spacing 0.75 mm, window level 70, window width 320. CT images of the ankle joint were obtained after scanning. In order to facilitate the subsequent calculation of the ankle motion angle model, the scanning range is less than 1/3 distal tibia. The scanned CT images are stored in JPG format.

### Establishment of the three-dimensional geometric model

Mimics 21.0 software was used to extract the obtained CT data. Based on the biological anatomy of the foot and ankle, the standard foot and ankle structure model containing the ankle joint and the surrounding bones was reconstructed. The obtained geometric model file was imported into Geomagic Wrap 2021, USA, for subdivision, noise reduction, smoothing, accurate surface, and other processes to form a three-dimensional solid foot and ankle model.

### Data measurement

**Fig. 1:**
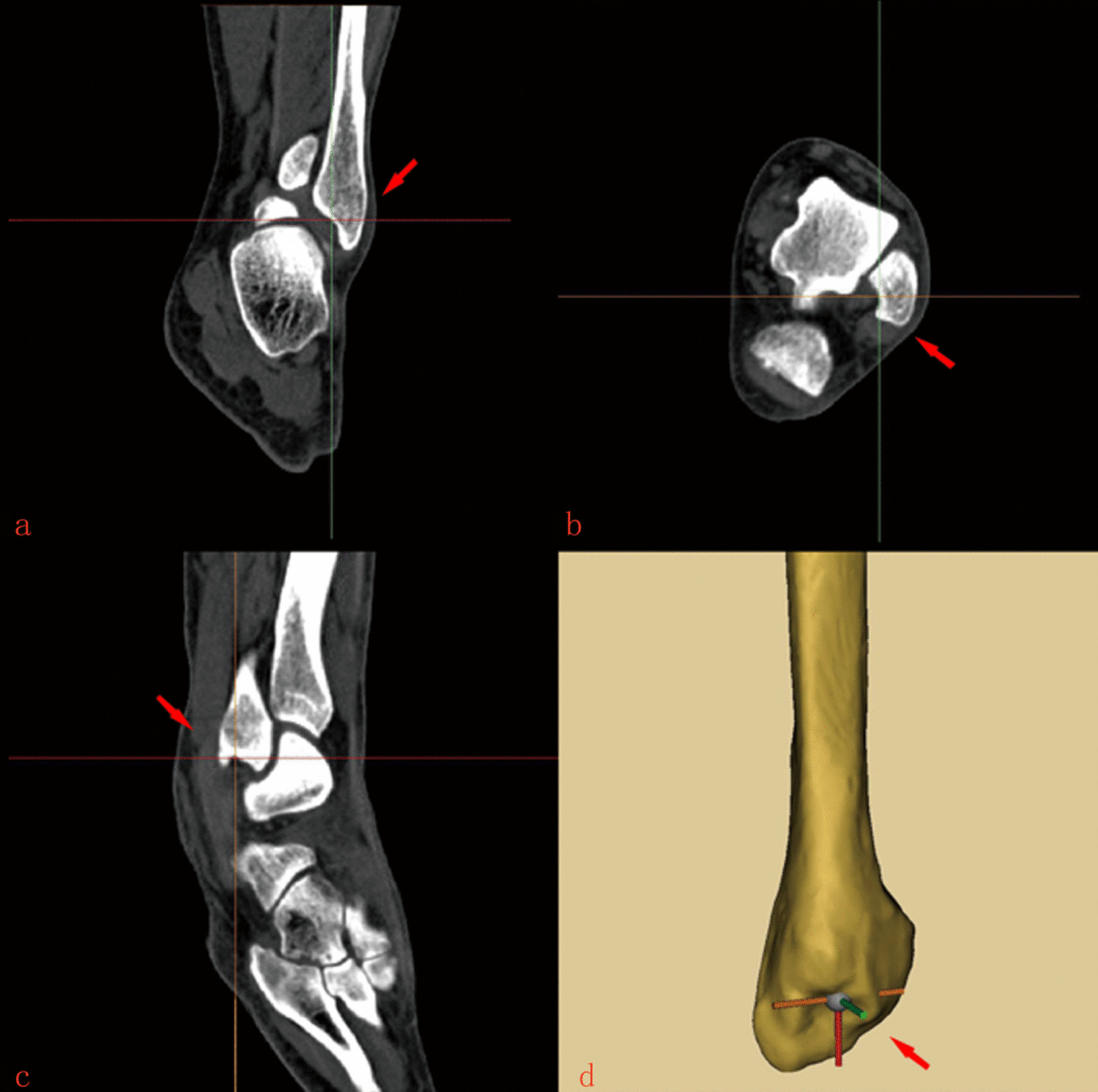
Lateral MF map after three-dimensional reconstruction. **a** The frontal plane of the lateral MF location. **b** The horizontal plane of the lateral MF location. **c** The sagittal plane of the lateral MF location. **d** Three-dimensional reconstruction of the location of the lateral MF

**Fig. 2:**
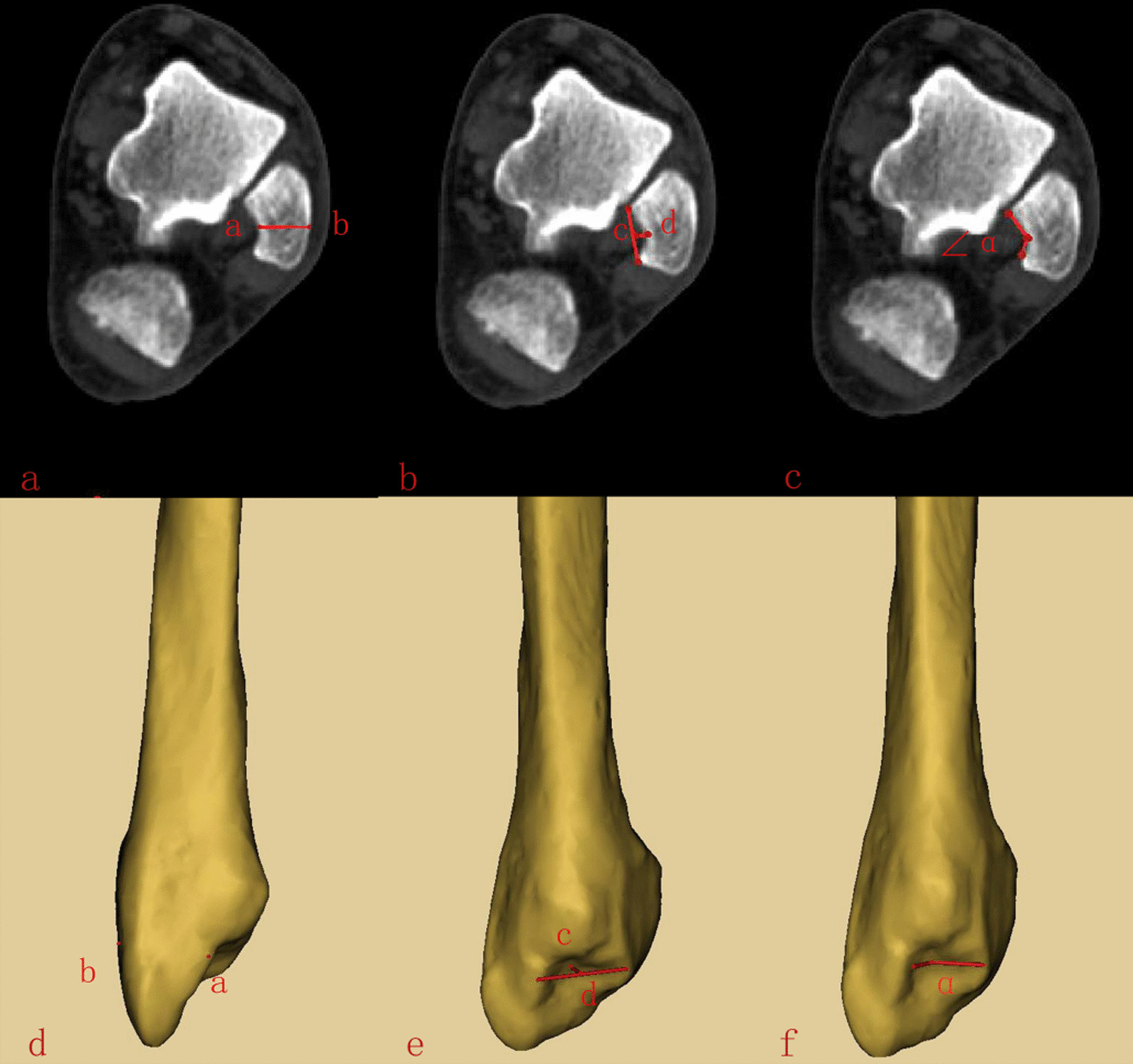
Cross-sectional data measurement diagram. **a** and **d** The deepest point of lateral malleolus fibula thickness. **b** and **e** The depth of lateral MF. **c** and **f** The angle of the MF

**Fig. 3:**
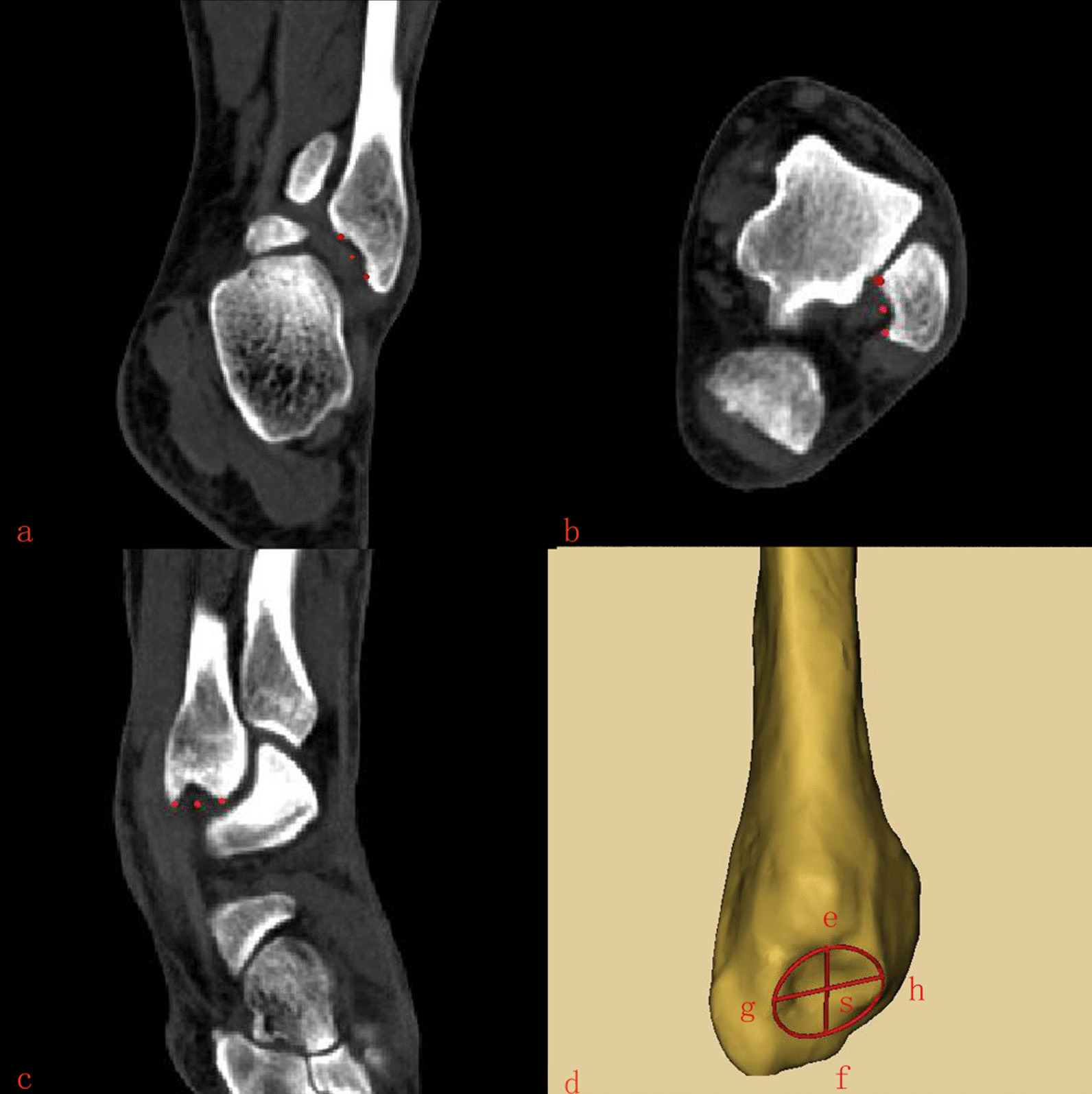
Localization and measurement of lateral MF area. 3a)The starting point of frontal area measurement. **b** The starting point of cross-sectional area measurement. **c** The starting point of sagittal area measurement. **d** ef: the length of the upper and lower surfaces of the lateral MF. gh: The left and right surface length of the outer lateral MF. s: Area size of lateral MF

The reconstructed model was re-imported into Mimics 21.0, and the deepest point of the lateral MF was located on the three-dimensional model, and then the morphological characteristics of the cross section were observed. The lateral MF was then carefully observed, and geometric parameters were measured. Inter-observer reliability was assessed by two authors independently. The measurement data are shown in Figs. 1, 2, and 3. We use the average length of each type ab as the radius to try to depict the safe area in the cross section. On the transverse position, the upper and lower edges of the lateral fibula were taken as the center of the circle, and ab was the radius to draw the area of mutual connection. Then, the intersection of the circle and the fibular cortical bone was connected to form a closed visual image on the cross section to represent the safe range of each type of lateral MF. Finally, the CT cross section of the C-type lateral MF was used to overlap the three types of visual images, and the safety range of the internal fixation of the lateral MF was compared.

### Statistical analyses

SPSS 26.0 software was used to perform statistical analysis on all the data. Since the MF is divided into three types, the expected incidence of each type is 33%. Assuming that the allowable error is 0.05, the expected sample size is 214 by the population rate formula. Measurement data were expressed as mean ± standard deviation. When the variance was uniform, the one-way variance was used for the analysis between groups, and Tamheini T2 (M) was used when the variance was unequal. The content of the analysis was whether there were significant differences in the measurement between the MF of different types. When *P* < 0.05, the difference was significant.

## Result

**Fig. 4:**
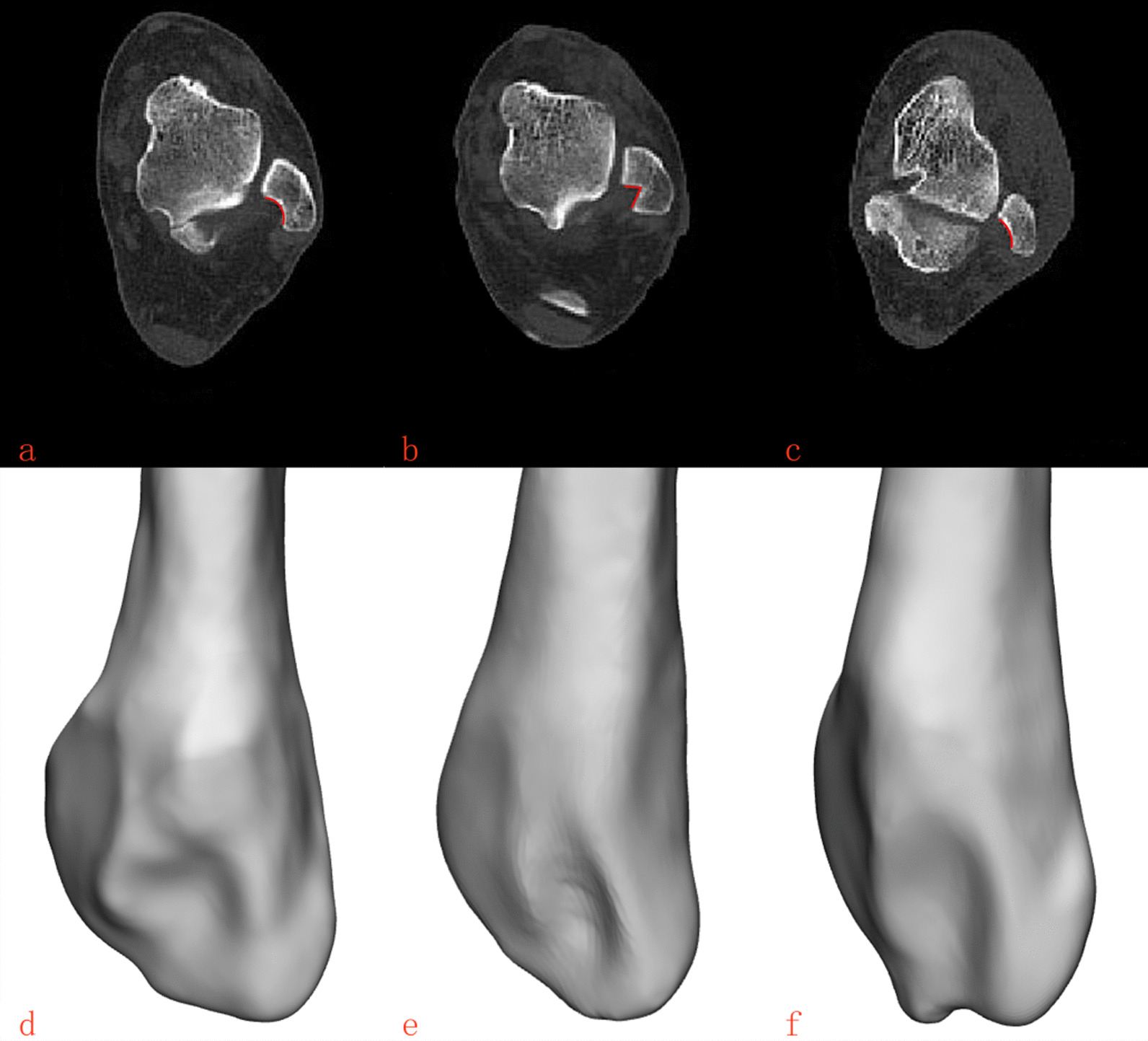
The classification of lateral MF. **a** and **d** Type C of the lateral MF. **b** and **e** Type V of the lateral MF. **c** and **f** Type Flat of the lateral MF

**Fig. 5:**
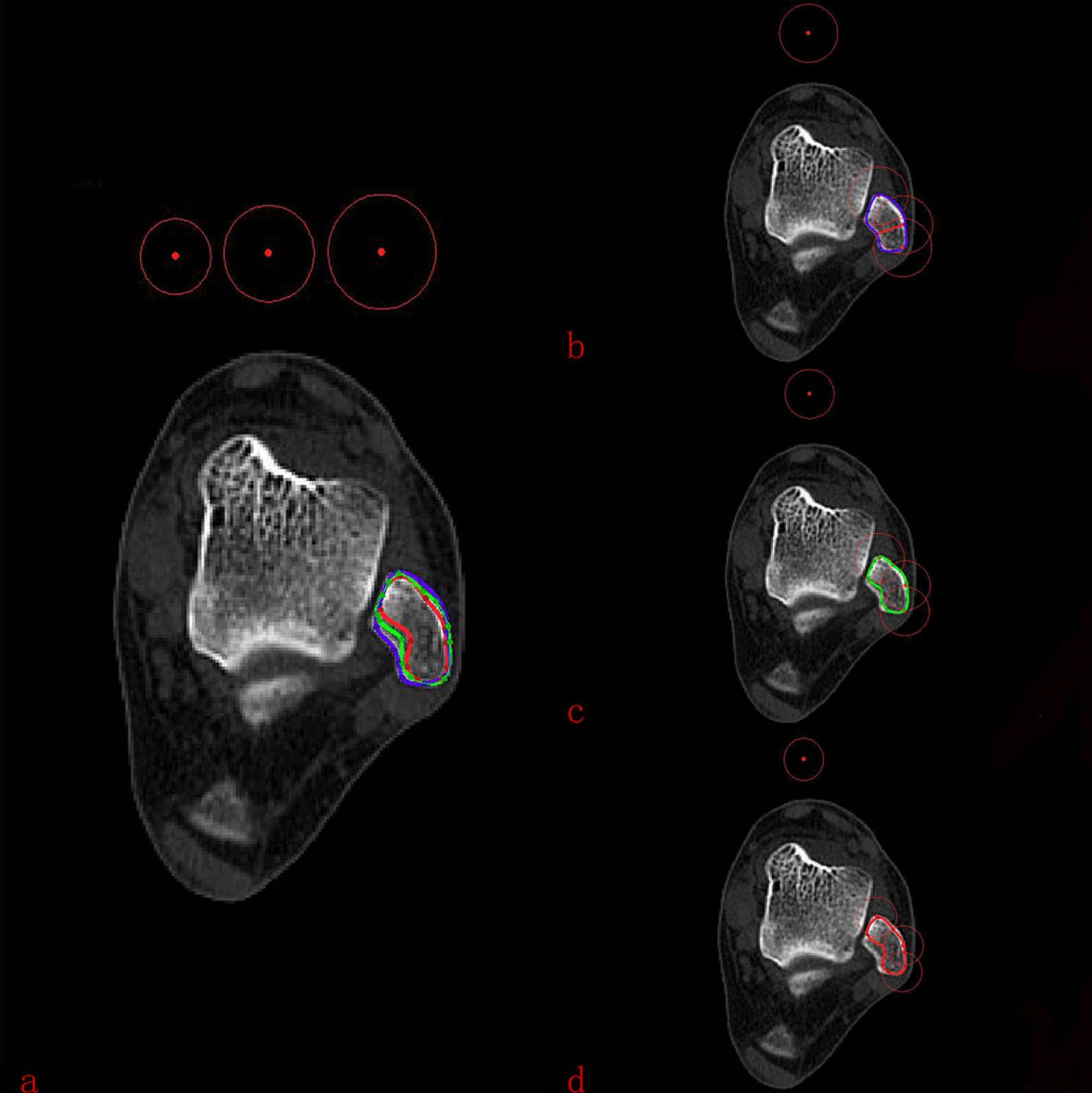
Simulate the three types of different safety zones when the screw is placed from the outside to the inside. **a** Three types in a unified cross section on their safe area. **b** F type safety zone. **c** C safety zone. **d** V type safety zone

According to the combination of three-dimensional reconstruction and cross section, the lateral MF could be divided into three types: type C, type V, and type Flat, As shown in Fig. 4. Among them, type C (107,43.1%) had the highest incidence, followed by type V (79, 32.2%). Apparently, type Flat (62, 24.7%) had the lowest proportion of all subjects. The geometric parameters corresponding to each type were measured, as shown in Table 1. Briefly, the deepest point of lateral malleolus fibular thickness in type V (3.98 ± 0.82) was significantly longer than that in type C (2.83 ± 0.54)and type Flat (1.84 ± 0.42). On the contrary, the lateral MF depth of Flat type (10.17 ± 0.77)was significantly longer than that of type C (9.69 ± 0.92)and type V (8.54 ± 0.84). The angle of the MF of Type Flat (136.31 ± 9.63) was the largest, followed by type C (116.51 ± 8.79), and type V (89.31 ± 9.07) was the smallest. (*P* < 0.05). No statistical differences were found in other parameters. Gender and feet of left and right did not affect the classification of the MF (*P* > 0.05), as shown in Tables 2 and 3. As shown in Fig. 5, The average thickness of the fibula of different types of lateral MF was used as the baseline to delineate the safe range. Then put the safety range on the same cross section, the type V area is the smallest, while the type Flat area is the largest.

**Table 1:** The geometric parameters corresponding to each type of the lateral MF

	Type C	Type V	type flat	In total	*F*	*P*
	107	79	62	248	–	–
ab (mm)	9.69 ± 0.92b	8.54 ± 0.84a	10.17 ± 0.77ab	9.44 ± 1.08	70.195	0.000
cd (mm)	2.83 ± 0.54b	3.98 ± 0.82a	1.84 ± 0.42ab	2.96 ± 1.04	196.557	0.000
ef (mm)	12.56 ± 1.92	12.32 ± 2.05	12.34 ± 2.16	12.43 ± 2.02	0.058	0.994
gh (mm)	8.51 ± 1.63	8.73 ± 1.73	8.65 ± 1.68	8.61 ± 1.66	0.382	0.683
<α(°)	116.51 ± 8.79b	89.31 ± 9.07a	136.31 ± 9.63ab	112.80 ± 20.08	479.020	0.000
S (mm^2^)	84.71 ± 23.67	83.49 ± 28.31	86.21 ± 29.87	84.20 ± 26.72	0.047	0.954

a versus Type C, *P* < 0.05; b versus Type V, *P* < 0.05

**Table 2:** Classification and measurement of the lateral MF of sex

Items	Male	Female	In total	*F*	*P*
	139	109	248	–	–
ab (mm)	9.52 ± 1.08	9.39 ± 1.07	9.44 ± 1.08	0.024	0.876
cd (mm)	3.03 ± 0.99	2.87 ± 1.10	2.96 ± 1.04	1.349	0.247
ef (mm)	12.57 ± 2.10	12.23 ± 1.90	12.43 ± 2.02	1.751	0.187
gh (mm)	8.61 ± 1.76	8.61 ± 1.54	8.61 ± 1.66	0.111	0.739
<α(°)	112.23 ± 18.86	113.55 ± 21.64	112.80 ± 20.08	0.262	0.609
S (mm^2^)	83.86 ± 26.58	85.67 ± 26.58	84.20 ± 26.72	0.027	0.870

**Table 3:** Classification and measurement of the lateral MF of feet of right and left

Items	Right	Left	In total	*F*	*P*
	137	111	248	–	–
ab (mm)	9.45 ± 1.05	9.43 ± 1.11	9.44 ± 1.08	0.170	0.895
cd (mm)	3.02 ± 0.99	2.89 ± 1.10	2.96 ± 1.04	1.349	0.247
ef (mm)	12.53 ± 2.06	12.29 ± 1.97	12.43 ± 2.02	0.882	0.349
gh (mm)	8.69 ± 1.67	8.53 ± 1.65	8.61 ± 1.66	0.555	0.457
<α(°)	112.41 ± 20.29	113.27 ± 19.89	112.80 ± 20.08	0.114	0.947
S (mm^2^)	83.43 ± 26.07	86.83 ± 25.06	84.20 ± 26.72	1.080	0.300

## Discussion

The lateral MF is on the posterolateral side of the lateral malleolus, which is the starting point of the posterior talofibular ligaments. We reconstructed 248 cases of the normal fibula and analyzed the anatomy of two-dimensional and three-dimensional images, respectively. Gandhi et al. [3] only measured the size and depth of the lateral MF without anatomical classification. We divided it into three types by the shape and angle of the transverse section of the lateral MF, among which type C has the most significant angle and relatively large surface area. The depth of the type V shape is the largest, indicating that the thickness of the lateral malleolus is small.

Gandhi et al. [3] found that 31.5% of the lateral fibula plate had the risk of invasion of the lateral MF, and the absence of a cross-sectional scan after surgery did not determine whether the lateral MF was invaded. Short-term pain may mask the pain caused by the invasion of the lateral MF. According to research, 20% of people have unexplained ankle pain one year after ankle fracture surgery [16, 17]. The lateral MF is the attachment point of the posterior talofibular ligament and then provides some space for ankle plantar flexion and valgus. The invasion of the lateral MF may lead to plantar flexion or valgus impingement syndrome, which eventually evolves into ankle osteoarthritis.

The authors speculate that the type of ankle fracture and inferior tibiofibular fixation may be the main causes of lateral MF invasion. For example, Lauge-Hansen supination external rotation, pronation abduction, and combined ankle fracture need internal fixation or multiple screw placement near lateral MF, which increases the risk of lateral MF invasion. In addition to the location and number of internal fixators, the important factor of lateral MF invasion may be the size of itself. Larger or deeper MF is more likely to be invaded. We found that the average area of the MF was 84.2 mm^2^, accounting for 80% of the medial area of the distal fibula; the average distance from the deepest point of the MF to the lateral fibula was about 2.96 mm, accounting for 32.7% of the fibula depth. We founded that in the same case, the greater the depth of the type V and the smaller the fibula thickness, the greater the risk of MF invasion. Therefore, the shape of the MF should be considered when the fixation screw is placed from the outside of the distal fibula. The type V should not exceed 8 mm; otherwise, a very large wind invades the lateral MF. If it is other types, no more than 10 mm screws can be selected. At the same time, in order to verify whether the implant depth was completely guaranteed not to invade the lateral malleolus fossa, we divide the safety range by the thickness of the fibula corresponding to the type V, type C, and Type Flat, which is 8 mm, 9 mm, and 10 mm, respectively. As shown in Fig. 5, when we put the three safety ranges on the same plane, we could saw that the type V is the smallest and the Type Flat is the largest. In clinical practice, when the internal fixation is implanted from the outside of the fibula, we observe the lateral MF before operation. If it is type V, the maximum depth of implantation at any angle outside the fibula can only be 8 mm. If it is the other two types, 9–10 mm can be selected, which is absolutely safe, but internal fixation instability may occur. After determining the type of the lateral MF, the most suitable depth can be selected when the lateral fibula is fixed, which can ensure the stability of the internal fixation and will not cause some unnecessary complications caused by the invasion of the lateral MF. In this study, such a length is absolutely safe. Another way to avoid damaging the lateral MF is to only go through the single cortex during implantation. Of course, selecting the most suitable implant is of great help to the healing of fractures.

This study has several limitations. Firstly, the sample size is small and it only has yellow people's information. Second, simple imaging data have certain errors, which may be more accurate in combination with anatomy. Future studies may be from anatomical or clinical research. This is a unique study in the medical literature—few studies on the classification of lateral MF. The clinical significance of the results requires further investigation. Analysis using imaging data alone needs to be more comprehensive.

## Conclusion

According to the morphology of the CT cross section, the lateral MF was divided into three types: type C, type V and type Flat. Type V is most likely to be invaded when fixing the distal fibula. Screws less than 9 mm should be selected when fixing, and screws no more than 10 mm should be selected when there are other types of lateral MF.

## Data Availability

The datasets used and analyzed during the current study are available from the first author on a reasonable request and upon completing a data sharing agreement. The data are not publicly available due to inclusion of unique health information from participants in a specific and limited region that could compromise participant confidentiality.
